# Kynurenic acid inhibits colon cancer proliferation in vitro: effects on signaling pathways

**DOI:** 10.1007/s00726-014-1790-3

**Published:** 2014-07-11

**Authors:** Katarzyna Walczak, Waldemar A. Turski, Grażyna Rajtar

**Affiliations:** 1 Department of Pharmacology, Medical University in Lublin, Chodźki 4a, 20-093 Lublin, Poland; 2 Department of Experimental and Clinical Pharmacology, Medical University in Lublin, Jaczewskiego 8, 20-090 Lublin, Poland

**Keywords:** Kynurenic acid, Colon cancer, Signaling pathways, PI3K/Akt pathway, MAPK, Wnt pathway

## Abstract

Kynurenic acid (KYNA), a tryptophan metabolite, inhibits proliferation of several cancer cell lines including colon cancer, renal cancer and glioblastoma cells. Previous studies reported that inhibitory properties of KYNA may be related to interactions of KYNA with cell cycle regulators and signaling proteins. However, the exact molecular interaction of KYNA with signaling pathways in colon cancer cells has not been studied to date. The molecular mechanism of KYNA activity towards colon cancer cells may be of great importance taking into consideration that KYNA is present in several tissues and physiological fluids, including gastrointestinal tract, and it is also present in various products of human diet. In this study, the inhibitory effect of KYNA on activation of phosphoinositide 3-kinase/Akt (PI3K/Akt) and mitogen-activated protein kinase (MAPK) signaling pathways in colon adenocarcinoma HT-29 cells was revealed. KYNA decreased phosphorylation of Akt, ERK 1/2 and p38 kinases in HT-29 cells. Interestingly, the study revealed also unexpected effect of KYNA on Wnt pathway in HT-29 cells. KYNA in millimolar concentrations increased protein expression of β-catenin. However, the nuclear translocation of β-catenin in HT-29 cells exposed to KYNA was not observed. Moreover, KYNA 1 mM increased antiproliferative properties of inhibitors of signaling pathways: wortmannin, PD98059, SB202190 and IWR-1. Taking into consideration these results, KYNA may be seen as a potential chemopreventive agent in colon cancer or supportive agent in standard cancer chemotherapy. However, the interactions between KYNA, Wnt signaling pathway and β-catenin need further studies to exclude potential effect of KYNA on colon carcinogenesis.

## Introduction

Kynurenic acid (KYNA), a metabolite of the kynurenine pathway of tryptophan degradation is known as a neuroprotective agent in central nervous system. However, KYNA was also found in physiological fluids in gastrointestinal tract: human saliva (Kuc et al. 2006), gastric juice, bile, pancreatic juice (Paluszkiewicz et al. 2009), mucus of rat small intestine (Kuc et al. 2008) as well as mucus of pig (Paluszkiewicz et al. 2009) and human colon (Walczak et al. 2011). KYNA may affect several various cellular processes as it is also present in several products of human diet (Turski et al. 2009, 2011) and it is absorbed after intragastric administration into the bloodstream and transported to peripheral organs (Turski et al. 2009). Although physiological role of KYNA in gastrointestinal tract in not fully studied, we found previously that KYNA may play a crucial role in cancer cell proliferation.

KYNA inhibited proliferation of several cancer cell lines including colon cancer Caco-2, HT-29, LS-180 cells (Walczak et al. 2011), renal cancer Caki-2 cells (Walczak et al. 2012b) and glioblastoma T98G cells (Walczak et al. 2014). There are only few data concerning the potential molecular mechanisms of antiproliferative activity of KYNA in cancer cells. The inhibitory properties of KYNA might be related to overexpression of cyclin-dependent kinases (CDK) inhibitor p21 Waf1/Cip1 (Walczak et al. 2012a, b). KYNA was also reported as a potential inhibitor of p38 kinase in renal cancer cells (Walczak et al. 2012b); however, the exact molecular interactions of KYNA with signaling pathways in colon cancer cells have not been studied to date.

Phosphoinositide 3-kinase/Akt (PI3K/Akt), mitogen-activated protein kinase (MAPK) and Wnt signaling pathways are crucial pathways involved in a control of proliferation of colon cancer cells (Inoki et al. 2002; Rubinfeld and Seger 2005; Fre et al. 2009). Signaling cascade, activated through subsequent phosphorylation of kinases, processes extracellular and intracellular signals into specific biological response including proliferation, invasiveness or neoplastic transformation (Chang and Karin 2001; Loesch et al. 2010). Thus, disturbances in those signaling pathways may lead to colon cancerogenesis or cancer progression. Moreover, Wnt pathway plays role in various crucial processes including growth and differentiation, organogenesis, migration, but also carcinogenesis. Wnt signaling pathway is also involved in intestinal morphogenesis and the maintenance of intestinal homeostasis (Fre et al. 2009).

The aim of this study was to reveal the potential interactions of KYNA with signaling pathways to determine its potential role as a chemopreventive or supportive agent in standard colon cancer therapy. Although several prognostic factors allow to classify the standard risk of patients with the same tumor entity, it is still not possible to precisely predict the individual response to standard anticancer therapies (Efferth 2012).

In this work, we show that KYNA inhibits the activation of Akt, extracellular signal-regulated kinases 1/2 (ERK 1/2) and p38 kinase and enhances β-catenin expression in HT-29 cells.

## Materials and methods

### Drugs

KYNA, wortmannin, PD98059, SB202190, IWR-1 were obtained from Sigma Aldrich (St. Louis, MO, USA). KYNA was dissolved in 1 N NaOH, and then phosphate buffered saline (PBS). Wortmannin, PD98059, SB202190 and IWR-1 were dissolved in dimethyl sulfoxide (DMSO); however, the final concentration of DMSO in samples was less than 0.2 %. In preliminary experiments, no significant influence of solvents on cancer cell proliferation and morphology was observed.

### Cell culture

Colon adenocarcinoma HT-29 cells were obtained from ECACC (European Collection of Cell Cultures, Centre for Applied Microbiology and Research, SP, UK). Cells were grown in 1:1 mixture of Dulbecco’s modified Eagle’s medium (DMEM) and nutrient mixture Ham F-12 supplemented with 10 % fetal bovine serum (FBS), penicillin (100 U/ml) and streptomycin (100 µg/ml) and were maintained in a humidified atmosphere of 95 % air and 5 % CO_2_ at 37 °C. All reagents were obtained from Sigma Aldrich.

### Western blot analysis

HT-29 cells were lysed in RIPA buffer [2 % NP40 (Tergitol), 0.5 % sodium deoxycholate, 0.1 % SDS, 1 mM EDTA, 1 mM EGTA, 1 mM Na_3_VO_4_, 20 mM NaF, 0.5 mM DTT, 1 mM PMSF, protease inhibitor mixture in PBS, pH 7.4] and centrifuged at 14,000×*g* for 10 min. Protein content in supernatants was determined by BCA Protein Assay Kit (Pierce Biotechnology, Rockford, USA). Supernatants were solubilized in sample buffer (30 % glycerol, 10 % SDS, 0.5 M Tris–HCl, pH 6.8, 0.012 % bromophenol blue, 5 % β-mercaptoethanol), and boiled for 5 min. For Western blotting, equal amounts of proteins were electrophoresed on 7–12 % SDS-PAGE and transferred to polyvinylidene difluoride (PVDF) membrane. After blocking for 1 h at room temperature with 5 % non-fat dry milk in tris-buffered saline–0.1 % Tween 20 (TBS-T), membranes were probed at 4 °C overnight with primary antibodies [p-Akt (Ser473), p-PTEN (phosphatase and tensin homolog) (Ser380), p-mTOR (mammalian target of rapamycin) (Ser4882), p-GSK3β (Ser9), p-ERK 1/2 (Thr202/Tyr204), p-p38 (Thr180/Tyr182) 1:1,000, β-actin 1:2,000; Cell Signaling Technology, Danvers, USA]. The membranes were then washed in TBS-T buffer and incubated with secondary antibody coupled to horseradish peroxidase (1:2,000 in 5 % non-fat milk in TBS-T; Cell Signaling Technology) for 1 h at room temperature and visualized by using enhanced chemiluminescence (Pierce Biotechnology). Serial exposures were made on Kodak BioMax Light film (Eastman Kodak Company, Rochester, NY, USA).

### Immunofluorescence

HT-29 cells plated on Lab-Tek Chamber Slides (Nunc) were allowed to grow for 24 h in a humidified atmosphere of 95 % air and 5 % CO_2_ at 37 °C. Cells were then treated with KYNA 5 mM for 24 h. After incubation, cells were washed with PBS, fixed with 3.7 % formaldehyde in PBS for 10 min and permeabilized with 0.2 % Triton-X100 in PBS for 7 min. After 30 min of treatment with 5 % BSA in PBS, the cells were exposed to primary antibodies against β-catenin (1:100; Cell Signaling Technology) overnight at 4 °C. Cells were then washed with PBS and incubated with secondary antibody conjugated with fluorescein isothiocyanate (FITC) (1:100) (Sigma Aldrich) for 2 h at room temperature. Cell images were captured with phase-contrast and fluorescence microscopy (Olympus BX51 System Microscope; Olympus Optical Co., Ltd., Tokyo, Japan, and CellFamily AnalySIS software) at 400× magnification.

### Proliferation assay (MTT assay)

HT-29 cells were plated on 96-well microplates (Nunc) at a density of 3 × 10^4^. Next day, the culture medium was removed and HT-29 cells were exposed to serial dilutions of KYNA (0.01, 0.1, 1 mM), wortmannin (1.5 µM), PD98059 (5 µM), SB202190 (2.5 µM), IWR-1 (1.5 µM) or combinations of these compounds with KYNA in a fresh medium supplemented with 10 % FBS. Cell proliferation was assessed after 96 h by using the MTT method in which the yellow tetrazolium salt [3-(4,5-dimethylthiazol-2-yl)-2,5-diphenyltetrazolium bromide, MTT] is metabolized by viable cells to purple formazan crystals. Tumor cells were incubated for 3 h with MTT solution (5 mg/ml). Formazan crystals were solubilized overnight in SDS (sodium dodecyl sulphate) buffer (10 % SDS in 0.01 N HCl), and the product was quantified spectrophotometrically by measuring absorbance at 570 nm wavelength using E-max Microplate Reader (Molecular Devices Corporation, Menlo Park, CA, USA).

### Data analysis

Data were presented as the mean value and standard error of the mean (SEM). Statistical analysis was performed using one-way ANOVA with Tukey post hoc test; *p* < 0.05 was considered statistically significant.

Western blots showed in the figures were selected as the most representative of the series of repetitions *n* ≥ 3. The Western blots were quantified by densitometry using NIH ImageJ software. The data were normalized relative to β-actin. The results of densitometric analysis are shown as percentage of control (the changes ≥20 % were considered as significant).

## Results

To investigate whether KYNA affects signaling pathways in colon cancer cells, we studied phosphorylation status of Akt and other elements of PI3K/Akt pathway, phosphorylation of ERK 1/2 and p38 kinases and protein expression of β-catenin in colon adenocarcinoma HT-29 cells treated with 1 mM concentration of KYNA for 5 min–48 h or treated with KYNA in the range of concentration 0.01–5 mM for 4, 24 and 48 h.

**Fig. 1:**
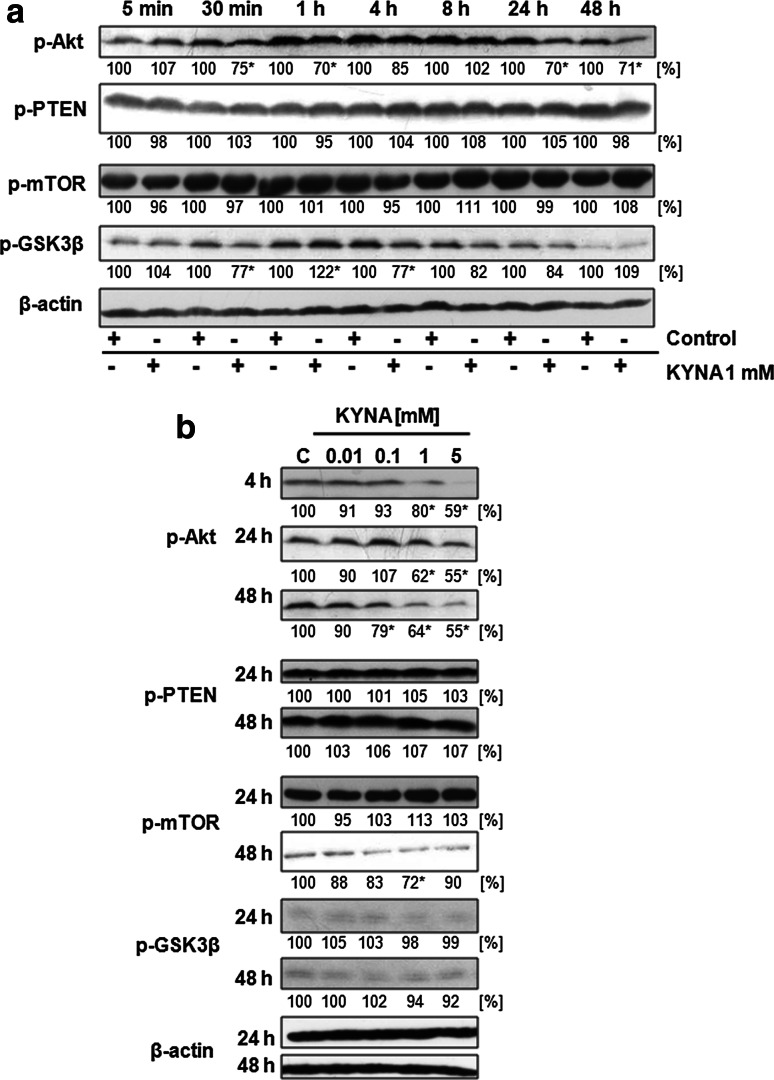
Effect of KYNA on activation of PI3K/Akt signaling pathway in HT-29 cells. Western blot analysis of phosphorylation status of selected proteins of PI3K/Akt signaling pathway in HT-29 cells after treatment with KYNA 1 mM for 5 min–48 h (**a**) and treatment with KYNA in the range of concentrations 0.01–5 mM for 4, 24 and 48 h or 24 and 48 h (**b**); (*C* control; not treated). Western blots shown in the figure were selected as the most representative of the series of repetitions *n* ≥ 3. The data were normalized relative to β-actin. The results of densitometric analysis are shown as % of control (the changes ≥20 % were considered as significant)

KYNA 1 mM inhibited phosphorylation of Akt kinase with the highest effectiveness after 30 min, 1, 24 and 48 h (Fig. 1a). Moreover, KYNA inhibited Akt activation in a dose-dependent manner (Fig. 1b). Furthermore, modulation of GSK3β phosphorylation was observed after incubation with KYNA 1 mM (Fig. 1a). However, KYNA did not affect phosphorylation status of mTOR and PTEN (Fig. 1a, b).

**Fig. 2:**
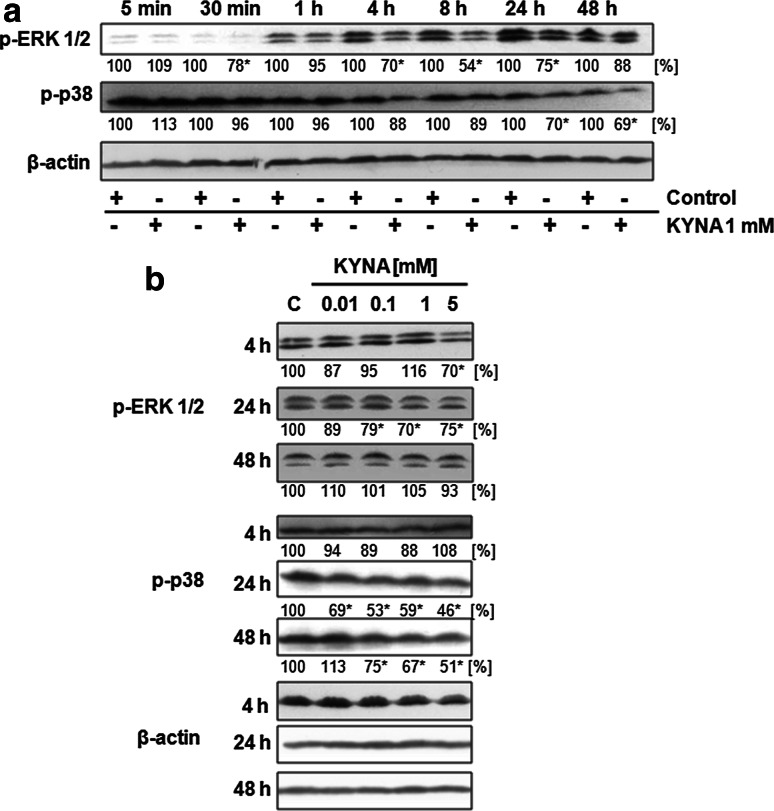
Effect of KYNA on activation of ERK 1/2 and p38 kinases in HT-29 cells. Western blot analysis of phosphorylation status of ERK 1/2 and p38 kinases in HT-29 cells after treatment with KYNA 1 mM for 5 min–48 h (**a**) and treatment with KYNA in the range of concentrations 0.01–5 mM for 4, 24 and 48 h (**b**); (*C* control; not treated). Western blots shown in the figure were selected as the most representative of the series of repetitions *n* ≥ 3. The data were normalized relative to β-actin. The results of densitometric analysis are shown as % of control (the changes ≥20 % were considered as significant)

The inhibitory effect of KYNA was also observed in activation of MAPK: ERK 1/2 and p38 kinases. KYNA 1 mM decreased phosphorylation of ERK 1/2 kinase after 4, 8 and 24 h of incubation (Fig. 2a). Decreased phosphorylation of ERK 1/2 was observed after 24 h incubation with 0.1, 1 and 5 mM KYNA (Fig. 2b). Additionally, inhibitory effect of KYNA was observed in p38 activation after 24 and 48 h of incubation (Fig. 2a). Decrease of p38 phosphorylation was reported after 24 h incubation with 0.01, 0.1, 1 and 5 mM KYNA and after 48 h incubation with 0.1, 1 and 5 mM KYNA (Fig. 2b).

**Fig. 3:**
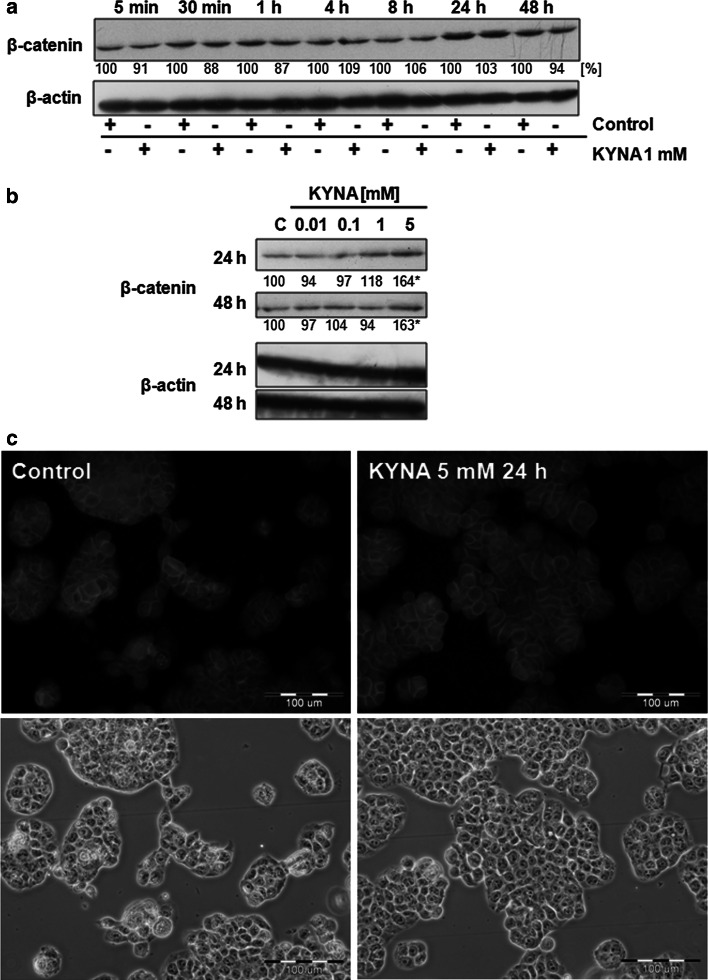
Effect of KYNA on β-catenin expression and cellular localization in HT-29 cells. Western blot analysis of protein expression of β-catenin in HT-29 cells after treatment with KYNA 1 mM for 5 min–48 h (**a**) or treatment with KYNA in the range of concentrations 0.01–5 mM for 24 and 48 h (**b**); (*C* control; not treated). Western blots shown in the figure were selected as the most representative of the series of repetitions *n* ≥ 3. The data were normalized relative to β-actin. The results of densitometric analysis are shown as % of control (the changes ≥20 % were considered as significant). **c** Immunofluorescent staining for β-catenin in HT-29 cells treated with KYNA 5 mM for 24 h (control; not treated). Magnification×400

To investigate the potential contribution of KYNA in activation of Wnt pathway, we studied the protein expression of one of the key protein in this pathway—β-catenin. Although there was no effect on β-catenin expression during incubation with KYNA 1 mM (Fig. 3a), higher concentration of KYNA increased expression of this protein after 24 and 48 h of incubation (Fig. 3b). However, no translocation of β-catenin was observed in HT-29 cells treated with KYNA 5 mM for 24 h (Fig. 3c).

**Fig. 4:**
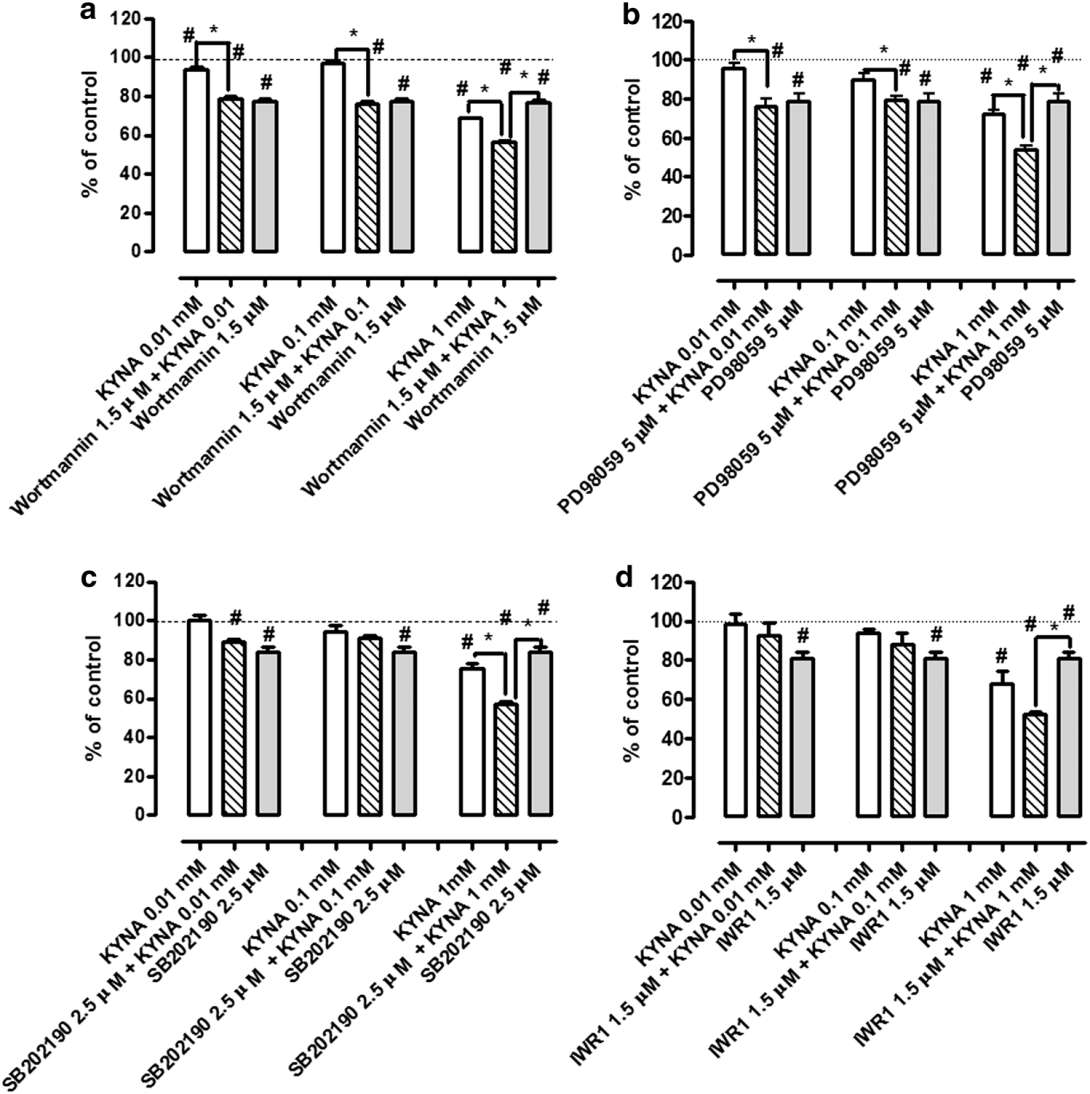
Effect of KYNA on antiproliferative activity of signaling pathway inhibitors in HT-29 cells. The effect of KYNA (0.01, 0.1, 1 mM) on antiproliferative properties of wortmannin (1.5 µM) (**a**), PD98059 (5 µM) (**b**), SB202180 (2.5 µM) (**c**) or IWR-1 (1.5 µM) (**d**) in HT-29 cells. Proliferation was assessed by MTT assay after 96 h of incubation. Data represent a mean value (% of control) ± SEM of six independent experiments. The control value (100 %) was marked by a *dotted line*. Star (*) statistically significant difference between groups marked graphically at *p* < 0.05, *Hash (#)* statistically significant difference vs. control at *p* < 0.05 (one-way ANOVA with Tukey post hoc test)

KYNA effect on the antiproliferative activity of inhibitors of signaling pathways was studied by means of MTT method. KYNA in 1 mM concentration inhibited proliferation of HT-29 cells to an average of 29 % after 96 h of incubation (Fig. 4). Concentrations of inhibitors of signaling pathways were chosen in preliminary experiments to inhibit proliferation of HT-29 cells by 15–25 %. KYNA 1 mM increased antiproliferative properties of wortmannin (1.5 µM), PD98059 (5 µM), SB202190 (2.5 µM) and IWR-1 (1.5 µM) by 21, 25, 27 and 28 %, respectively (Fig. 4a–d). KYNA in concentrations of 0.01 and 0.1 mM did not affect antiproliferative properties of the tested inhibitors (Fig. 4a–d).

## Discussion

In this study, the inhibitory effect of KYNA on the activation of PI3K/Akt and MAPK signaling pathways in colon adenocarcinoma HT-29 cells was revealed. KYNA decreased phosphorylation of Akt, ERK 1/2 and p38 kinases. The molecular mechanism of KYNA activity towards colon cancer cells may be of great importance taking into consideration that KYNA is not only present in several tissues and physiological fluids, including gastrointestinal tract (Kuc et al. 2006, 2008; Paluszkiewicz et al. 2009; Walczak et al. 2011), but it is also a component of food products such as vegetables, honeybee products and medicinal herbs (Turski et al. 2009, 2011). Moreover, Turski et al. (2009) reported that KYNA administered intragastrically in rats was absorbed from the intestine into the bloodstream and transported to liver and kidney in a time- and dose-dependent manner.

Previous studies indicated antiproliferative properties of KYNA towards colon adenocarcinoma HT-29, Caco-2 and LS-180 cells (Walczak et al. 2011, 2012a), renal cell carcinoma Caki-2 cells (Walczak et al. 2012b) and glioblastoma T98G cells (Walczak et al. 2014). However, the exact molecular mechanism of antiproliferative activity of KYNA has not been fully revealed. Disturbances in the signaling pathways may lead to colon cancerogenesis or cancer progression and permanent activation of signaling kinases has been observed in several cancer cells including colon cancer cells. In this study, the inhibitory effect of KYNA on the activation of Akt, ERK 1/2 and p38 kinases was revealed in colon cancer HT-29 cells. KYNA decreased phosphorylation of p38 kinase in HT-29 cells after 24 and 48 h of incubation with tested compound. The inhibitory effect of KYNA on p38 kinase activation has been already reported in renal cancer Caki-2 cells (Walczak et al. 2012b). p38 kinase is a member of the family of MAPK, serine/threonine-specific protein kinases, which plays a crucial role in regulation of growth, differentiation, motility and apoptosis (Chang and Karin 2001; Loesch et al. 2010). p38 MAPK pathway is activated in response to various stress factors including osmotic shock, cytokines, lipopolysaccharide, UV radiation and growth factors. It plays crucial role in several biological processes such as inflammatory responses, differentiation, proliferation and apoptosis (Holloway and Coulson 2006; Kyriakis and Avruch 2001). p38 MAPK pathway, regulating transcription of genes coding cytokines, transcription factors and receptors, is also involved in the control of cell cycle (Zarubin and Han 2005).

However, the inhibitory effect of KYNA on other member of MAPK, ERK 1/2 kinase has not been reported in cancer cells. In this study, we revealed that KYNA inhibited phosphorylation of ERK 1/2 kinase in HT-29 cells from 4 to 24 h of incubation with tested compound. ERK pathway is involved in cellular processes such as survival and proliferation. Its disturbances may lead to cancerogenesis, and thus, ERK pathway is an important target in the cancer treatment (Halilovic and Solit 2008). ERK pathway is activated in response to various extracellular signals including mitogens, growth factors and cytokines, through activation of kinase cascade Ras/Raf/MEK 1/2/ERK 1/2/p90RSK (Rubinfeld and Seger 2005).

In this study, we revealed that KYNA inhibited also PI3K/Akt pathway in HT-29 cells by decreasing phosphorylation of Akt kinase. PI3K/Akt pathway is activated by growth factors and is involved in proliferation (Inoki et al. 2002), cell cycle regulation (Gesbert et al. 2000; Zhou et al. 2001), apoptosis (Duronio 2008) and glycogen synthesis (Hajduch et al. 2001). Disturbances in this signaling pathway may lead to cancerogenesis or cancer progression (Liu et al. 2009; Saal et al. 2007). Akt kinase phosphorylates various target proteins including transcription factors NFκB and FOXO-3, pro-apoptosis Bad protein, caspase-9, MDM2, kinases c-Raf and GSK3β (Franke et al. 1997; Burgering and Coffer 1995; Duronio 2008; Liu et al. 2009). Akt is also a negative regulator of CDK inhibitors p21 Waf1/Cip1 and p27 Kip1 (Gesbert et al. 2000; Zhou et al. 2001). The results suggested that Akt inhibition in KYNA-treated HT-29 cells might result in previously reported overexpression of p21 Waf1/Cip1 (Walczak et al. 2012a). Interestingly, KYNA modulated also phosphorylation of GSK3β in HT-29 cells. However, the results suggested that there was no correlation between GSK3β modulation and inhibition of Akt kinase in HT-29 cells. Akt kinase phosphorylates GSK3β kinase which leads to its inactivation and thus, prevents inhibition of glycogen synthase and activates glycogen synthesis (Manning and Cantley 2007). As GSK3β kinase is involved in several cellular processes and its activation may be dependent on other elements (Welcker et al. 2003; Yeh et al. 2004; Wei et al. 2005; Sundqvist et al. 2005), it cannot be excluded that the modulation of GSK3β phosphorylation in HT-29 cells is the result of KYNA interaction with other signal elements or cell metabolism. Moreover, no effect on phosphorylation of other elements of PI3K/Akt pathway: PTEN phosphatase and mTOR kinase were observed in KYNA-treated HT-29 cells.

KYNA decreased phosphorylation of Akt, ERK 1/2 and p38 kinases inhibiting activation of PI3K/Akt and MAPK signaling pathways in HT-29 cells, which may lead to inhibition of colon cancer cell proliferation. Interestingly, the study revealed also unexpected effect of KYNA on Wnt pathway in HT-29 cells. KYNA in high millimolar concentrations increased protein expression of β-catenin after 24 and 48 h. Wnt pathway plays role in various crucial physiological processes, but also carcinogenesis. It is also involved in intestinal morphogenesis and intestinal homeostasis: its inhibition leads to the arrest of proliferative processes in the region of intestinal crypts (Fre et al. 2009). A main protein effector of Wnt signaling cascade is β-catenin, which is also involved in cell adhesion. Whether KYNA stimulates expression of this protein or affects its stability in colon cancer HT-29 cells needs to be elucidated. It cannot be excluded that, in some conditions, KYNA interactions with Wnt signaling pathway may lead to stimulation of colon cancer cell proliferation. However, we did not observe nuclear translocation of β-catenin in HT-29-treated cells, which may suggest that β-catenin is involved rather in the processes of adhesion than proliferation.

To verify which signaling pathway might be mainly responsible for antiproliferative activity of KYNA, HT-29 cells were exposed to selected inhibitors of signaling pathways and KYNA. Importantly, KYNA increased antiproliferative properties of inhibitors of PI3K/Akt (wortmannin), ERK (PD98059), p38 MAPK (SB202190) and Wnt (IWR-1) signaling pathways, which confirmed that it may affect several cellular processes and thus, inhibit proliferation of HT-29 cells. Antiproliferative activity of KYNA may be a result of diverse interactions with various signaling pathways and cell cycle regulators in colon cancer cells. Additionally, previous studies suggested a potential role of receptors, especially glutamate receptors, in antiproliferative properties of KYNA. KYNA is an antagonist of ionotropic glutamate receptors, agonist of G-protein coupled receptor 35 (GPR35) (Wang et al. 2006) and aryl hydrocarbon receptor (AhR) (DiNatale et al. 2010). Presence of glutamate receptors in cancer cells and antiproliferative properties of antagonists of glutamate receptors towards several cancer cell lines were previously reported (Rzeski et al. 2001; Stepulak et al. 2005). It was also shown that there is a functional relationship between the subunits of glutamate receptors and intracellular biochemical pathways regulating cell proliferation, invasion and metastasis in cancer cells (Luksch et al. 2011). Although there are limited data concerning contribution of other receptors in cancer cell proliferation, the involvement of GPR35 receptors in antiproliferative activity of KYNA against colon cancer cells cannot be excluded. These receptors are predominantly expressed in the gastrointestinal tract and in immune cells (Wang et al. 2006). On the other hand, AhR receptors are involved in several processes including cell proliferation, apoptosis, tumor suppression and others (reviewed in Fujii-Kuriyama and Kawajiri 2010). Although KYNA is considered as an agonist of this receptor (DiNatale et al. 2010), in certain conditions its biological impact on cellular processes is similar to the biological activity of AhR antagonist, such as resveratrol (Maaetoft-Udsen et al. 2012). On the other hand, the observed changes may be also a result of the KYNA effect on cancer cell metabolism. Previous studies revealed that KYNA decreased ATP synthesis in the presence of glutamate and malate in mitochondria isolated from rat heart (Baran et al. 2001).

Taking into consideration these results, KYNA may be seen as a potential chemopreventive agent in colon cancer or supportive agent in standard cancer chemotherapy. Importantly, in vivo studies revealed that administration of KYNA intravenously in the dose of 50 or 100 mg/kg/h in rats was well tolerated (Marciniak and Turski 2010). Moreover, KYNA is present in several food products (Turski et al. 2009, 2011) and is absorbed after intragastric administration to blood stream (Turski et al. 2009). These findings further support the hypothesis of the potential therapeutic implications of KYNA in cancer therapy.

On the other hand, there are some ambiguous data concerning the role of KYNA in the process of carcinogenesis, which should be clarified in further in vivo studies. Elevated KYNA concentration was observed in mucus aspirated from human ceacum and colon ascendens from patients diagnosed with colon cancer (Walczak et al. 2011). Although it may be the result of increased metabolism of cancer cells, the potential role of KYNA in colon carcinogenesis should be elucidated. Moreover, taking into consideration that disturbances in expression of β-catenin were observed in 40 % of patients diagnosed with colon cancer (Pasz-Walczak et al. 2004), the biological role of overexpressed β-catenin in KYNA-treated HT-29 cells should be also clarified.

## Abbreviations

AhR: Aryl hydrocarbon receptor
CDK: Cyclin-dependent kinases
DMEM: Dulbecco’s modified Eagle's medium
DMSO: Dimethyl sulfoxide
ECACC: European Collection of Cell Cultures
ERK: Extracellular signal-regulated kinases
FBS: Fetal bovine serum
FITC: Fluorescein isothiocyanate
GPR35: G-protein coupled receptor 35
KYNA: Kynurenic acid
MAPK: Mitogen-activated protein kinase
mTOR: Mammalian target of rapamycin
MTT: (3-(4,5-Dimethylthiazol-2-yl)-2,5-diphenyltetrazolium bromide)
PBS: Phosphate buffered saline
PI3K: Phosphoinositide 3-kinase
PTEN: Phosphatase and tensin homolog
PVDF: Polyvinylidene difluoride
SDS: Sodium dodecyl sulphate
SEM: Standard error of the mean
TBS: Tris-buffered saline
